# Molecular Genetics of Conjunctival Melanoma and Prognostic Value of *TERT* Promoter Mutation Analysis

**DOI:** 10.3390/ijms22115784

**Published:** 2021-05-28

**Authors:** Natasha M. van Poppelen, Jolique A. van Ipenburg, Quincy van den Bosch, Jolanda Vaarwater, Tom Brands, Bert Eussen, Frank Magielsen, Hendrikus J. Dubbink, Dion Paridaens, Erwin Brosens, Nicole Naus, Annelies de Klein, Emine Kiliç, Robert M. Verdijk

**Affiliations:** 1 Department of Ophthalmology, Erasmus MC University Medical Center, Doctor Molewaterplein 40, 3015 GD Rotterdam, The Netherlands; n.vanpoppelen@erasmusmc.nl (N.M.v.P.); j.vaarwater@erasmusmc.nl (J.V.); t.brands@erasmusmc.nl (T.B.); b.eussen@erasmusmc.nl (B.E.); d.paridaens@oogziekenhuis.nl (D.P.); n.naus@erasmusmc.nl (N.N.); e.kilic@erasmusmc.nl (E.K.); 2 Department of Clinical Genetics, Erasmus MC University Medical Center, Doctor Molewaterplein 40, 3015 GD Rotterdam, The Netherlands; f.magielsen@erasmusmc.nl (F.M.); e.brosens@erasmusmc.nl (E.B.); a.deklein@erasmusmc.nl (A.d.K.); 3 Department of Pathology, Section Ophthalmic Pathology, Erasmus MC University Medical Center, Doctor Molewaterplein 40, 3015 GD Rotterdam, The Netherlands; j.vanipenburg@erasmusmc.nl (J.A.v.I.); q.vandenbosch@erasmusmc.nl (Q.v.d.B.); h.dubbink@erasmusmc.nl (H.J.D.); 4 Department of Pathology, Radboud University Medical Center, Geert Grooteplein Zuid 10, 6525 GA Nijmegen, The Netherlands; 5 Department of Ocular Oncology, The Rotterdam Eye Hospital, Schiedamse Vest 180, 3011 BH Rotterdam, The Netherlands; 6 Department of Pathology, Leiden University Medical Center, Albinusdreef 2, 2333 ZA Leiden, The Netherlands

**Keywords:** conjunctiva, melanoma, molecular medicine, prognosis, *TERT* promotor mutation

## Abstract

The aim of this study was exploration of the genetic background of conjunctival melanoma (CM) and correlation with recurrent and metastatic disease. Twenty-eight CM from the Rotterdam Ocular Melanoma Study group were collected and DNA was isolated from the formalin-fixed paraffin embedded tissue. Targeted next-generation sequencing was performed using a panel covering *GNAQ*, *GNA11*, *EIF1AX*, *BAP1*, *BRAF*, *NRAS*, *c-KIT*, *PTEN*, *SF3B1*, and *TERT* genes. Recurrences and metastasis were present in eight (29%) and nine (32%) CM cases, respectively. *TERT* promoter mutations were most common (54%), but *BRAF* (46%), *NRAS* (21%), *BAP1* (18%), *PTEN* (14%), *c-KIT* (7%), and *SF3B1* (4%) mutations were also observed. No mutations in *GNAQ*, *GNA11*, and *EIF1AX* were found. None of the mutations was significantly associated with recurrent disease. Presence of a *TERT* promoter mutation was associated with metastatic disease (*p*-value = 0.008). Based on our molecular findings, CM comprises a separate entity within melanoma, although there are overlapping molecular features with uveal melanoma, such as the presence of *BAP1* and *SF3B1* mutations. This warrants careful interpretation of molecular data, in the light of clinical findings. About three quarter of CM contain drug-targetable mutations, and *TERT* promoter mutations are correlated to metastatic disease in CM.

## 1. Introduction

Conjunctival melanoma (CM) comprises 5–10% of all ocular melanoma [1,2,3]. The majority derives from primary acquired melanosis with atypia (PAM), but infrequently, CM develops from a pre-existing nevus or de novo [1,3,4,5,6]. CM has an incidence of 0.2–0.8 per million [3,6,7], with an increasing trend [3,8]. The 5- and 10-years cumulative incidence of CM-related mortality is 17–31% and 22–59%, respectively [5,7,9,10,11]. The prognosis of ocular melanoma, including CM and uveal melanoma (UM), depends on clinical and histopathological features, as well as the molecular genetic make-up [3,12,13]. During the past decade, the molecular make-up of UM has been well-characterized, with UM harboring recurrent mutations in guanine-nucleotide-binding protein-Q (*GNAQ*), guanine-nucleotide-binding protein-alpha 11 (*GNA11*), BRCA-associated protein 1 (*BAP1*), splicing factor 3 subunit 1 (*SF3B1*), and eukaryotic translation initiation factor 1A (*EIF1AX*). *BAP1* and *SF3B1* mutations are associated with the development of metastasis in UM. After the diagnosis of metastatic disease, patients with UM have a survival between 2–9 months [12]. When CM has metastasized, there are also very limited treatment options [1,13]. Yet, although CM as well as UM are ocular melanoma, CM certainly do show overlapping features, including molecular abnormalities with cutaneous melanoma [1,3,6,13,14]. For example, in 25–40% of the CM driver v-raf murine sarcoma, viral oncogene homolog B1 (*BRAF*) V600E/K mutations are described [1,2,6,13,15]. This incidence is higher as compared to other mucosal melanoma, which harbor a *BRAF* mutation in only 12% of cases. Although a correlation between *BRAF* mutations and poor prognostic factors has been described in cutaneous melanoma, no predictive value is yet reported for mucosal melanoma [16,17]. Other genes in which mutations have been identified in CM are the neuroblastoma RAS viral oncogene homolog (*NRAS*), Kirsten RAS oncogene homolog (*KRAS*), neurofibromin 1 (*NF1*), telomerase reverse transcriptase (*TERT*), tyrosine protein kinase (*c-KIT*), *TP53*, and *BAP1* [3,6,15,18]. Mutations in *GNAQ*/*GNA11* have also been described, but these are not the known activating hotspot mutations at amino acid Q209 or R183, which occur in UM [15,19]. The genetic background of the melanoma originating from these different locations, emphasizes the differences between UM and CM, and the similarities between CM and cutaneous melanoma. Furthermore, in contrast to UM, some of the mutations frequently found in CM are amenable to targeted therapies. However, the prognostic value of these molecular abnormalities in CM is largely unclear. The aim of this study was to further elucidate the genetic background of CM within the spectrum of melanoma and to correlate these findings with the development of recurrences and metastasis.

## 2. Results

### 2.1. Clinical and Histopathological Characteristics

Clinical and histopathological characteristics are listed in Table 1. Based on the availability of sufficient formalin-fixed paraffin-embedded (FFPE) tissue for DNA isolation, twenty-eight cases could be included. Gender was equally divided with 50% males and 50% females. The median age at the time of diagnosis was 64 years (range 16–89 years). Based on the clinical information, most tumors were (at least partly) located on the bulbar conjunctiva (16 cases, 57%) with involvement of the palpebral conjunctiva in 10 cases (36%), the fornix in 5 cases (18%), and the caruncle in 1 case (4%). The tumors had a median diameter of 0.7 cm (range 0.05–1.8 cm), with a median tumor thickness of 3.0 mm (range 0.18–7.70 mm). According to the Eighth Edition of the American Joint Committee on Cancer (AJCC) Cancer Staging [20], twelve cases (43%) were pathological tumor (pT) stage pT1, including six pT1a cases (21%) and five pT1b cases (18%), and thirteen cases were pT2 cases (46%), comprising one pT2a case (4%), eleven pT2b cases (39%), and two cases (7%) with unknown tumor thickness. In three cases (11%), the pT status was unknown. In eighteen cases (64%), the melanoma were derived from PAM, four melanoma (14%) developed from a nevus, and three melanoma (11%) were de novo lesions. In three cases (11%), the origin could not be reliably determined, based on the pathology reports and the available clinical information. 

Local recurrent disease occurred in eight patients (29%), between 6.8–156.8 months (median 29.3 months) after treatment. Nine patients (32%) developed metastatic disease between 1.7–49.2 months (median 14.3 months). Metastatic sites included lymph nodes (solitary or within the parotid gland) in all patients (n = 9), with metastatic disease in the orbit (n = 1), thyroid (n = 1), breast (n = 1), lung (n = 1), brain (n = 1), and spleen (n = 1). The thyroid and breast metastases were present in one patient, and the orbit and brain metastases were identified in one patient as well. The spleen and brain metastases were not histologically confirmed. The mean overall survival was 77.4 months (range 3.85–257.2 months), with a median of 62.8 months.

### 2.2. Mutation Analysis

**Figure 1:**
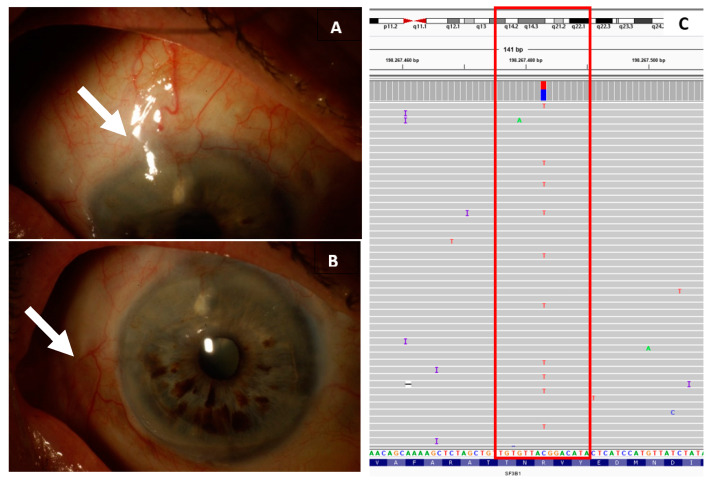
Clinical pictures and molecular data concerning the conjunctival melanoma harboring a *SF3B1* mutation. In (**A**) the macroscopic view of the melanoma located on the bulbar conjunctiva, within (**B**) the primary acquired melanomsis with atypia component (white arrow). Depicted in the red box in (**C**) is the molecular data concerning a p.Arg625His mutation in *SF3B1*, with an allele frequency of 42%, using the Integrative Genomics Viewer.

The specific mutations found per case are listed in Supplementary Table S1, with a summary of the mutations including correlation with metastatic and recurrent disease in Table 2. Fifteen CM cases (54%) showed a *TERT* promoter mutation. A mutation in the *BRAF* gene was identified in thirteen CM (46%), mostly affecting amino acid V600. *NRAS* mutations were seen in six cases (21%) and mutations in *BAP1* were identified in five CM (18%). A *PTEN* mutation was found in four CM (14%), and in two CM (7%), a mutation in *c*-*KIT* was identified. Interestingly, a p.Arg625His mutation in *SF3B1* was detected in one CM (4%). The diagnosis was unequivocally a CM in terms of both clinical and pathological reports. It was located in the nasal superior in the bulbar conjunctiva (Figure 1). None of the CM cases carried a mutation of *GNAQ*, *GNA11*, or *EIF1AX*.

**Figure 2:**
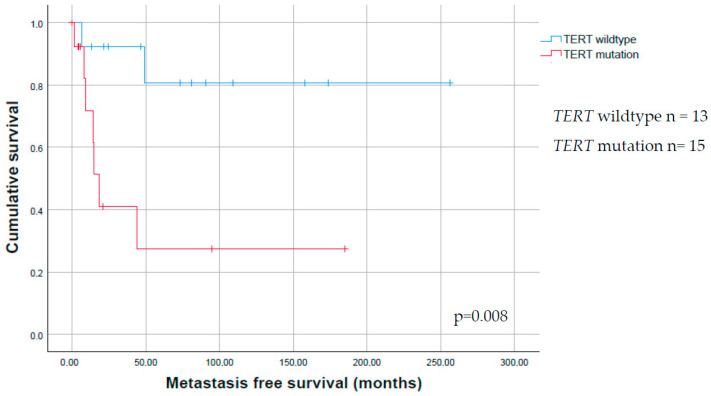
Kaplan–Meier survival estimate for the presence of a *TERT* promoter mutation in conjunctival melanoma. Kaplan–Meier survival estimate for the time to metastasis of conjunctival melanoma (CM), showing that patients with a CM with a *TERT* promoter mutation are more likely to develop metastatic disease.

The metastasis-free survival (MFS) of patients with a *TERT* promoter mutation was significantly shorter as compared to patients without a *TERT* promoter mutation in the tumor (*p* = 0.008, Table 2, Figure 2). No correlation between metastasis-free survival and mutation status of *BRAF*, *BAP1*, *SF3B1*, *NRAS*, *c-KIT*, and *PTEN* could be observed. 

No correlation was found between the presence of any mutations and the development of recurrences (Table 2). We also analyzed whether the mutations were correlated with sex, age, location (bulbar only versus involvement of the palpebral/caruncular/forniceal conjunctiva), pT status (pT1 versus pT2), tumor thickness, origin (PAM-derived melanoma versus non-PAM-derived melanoma). We did find an association between the presence of a *TERT* promoter mutation and the origin of the lesion (*p*-value = 0.005), with most cases (54%) developing either de novo or from a melanocytic nevus (Table 3).

### 2.3. Immunohistochemistry

In five CM cases that revealed a *BAP1* mutation using molecular testing, there was enough material available for testing the presence of a *BAP1* mutation using immunohistochemistry. Four of these cases did not show loss of expression of *BAP1* using immunohistochemistry, while one CM case did show loss of expression using *BAP1* immunohistochemistry, with presence of positive (internal) control tissue. 

## 3. Discussion

Pathways involved in the pathogenesis of CM included the MAPK/ERK pathway and the PI3K/AKT pathways, and these pathways overlap with the pathways involved in cutaneous melanoma [6].

The mutation that we found most frequent in CM is a *TERT* promoter mutation, congruent with other studies concerning ocular melanoma [6,13,14] and cancer originating from other sites. These mutations result in a new consensus binding site for E-twenty-six (ETS) transcription factors and this may contribute to increased *TERT*. The ETS transcription factors are downstream targets of the RAS-RAF-MAPK pathways, and *TERT* promoter mutations are suggested to have synergistic effects with activating *BRAF* or *NRAS* mutations to promote tumor cell proliferation [21]. *TERT* is involved in the AKT pathway, and plays an important role in cellular immortality [6]. *TERT* mRNA overexpression does not completely explain all effects of the *TERT* promoter mutations in tumorigenesis, and the role of immunohistochemistry in determining the *TERT* status is still a topic of debate [22]. Consequently, other undefined or epigenetic mechanisms of *TERT*-upregulating are expected to exist [21,23,24]. While a *TERT* promoter mutation is not found in conjunctival nevi, it is found in both PAM [14] and CM [6,14], with increased *TERT* expression leading to tumor progression [6]. In this context, the C>T or CC>TT nucleotide changes in these mutations are of interest, since this is the typical UV signature, in line with the UV-exposed location of most CM, as seen in our study and as compared to the molecular make up of cutaneous melanoma [6]. UM usually do not harbor mutations in or near the *TERT* gene [14,18,25]. It indicates that different pathways are involved in the development of CM and UM, as is also suggested by the differences in the presence of mutations in *BRAF*, *NRAS*, and *GNAQ*/*GNA11*. Since *TERT* promoter mutations are relatively common in CM, these mutations are of special interest with respect to clinical consequences. We did not find a correlation between the presence of any of the investigated mutations in this study and the well-known adverse histopathological parameters, as has been described for cutaneous melanoma, such as increasing tumor thickness and more advanced pT stage [26]. Previous studies reported an association between PAM with atypia and PAM-derived melanoma, with the presence of a *TERT* promoter mutation [13,14]. Remarkably, in the current study, we found a significant association with the presence of a *TERT* promoter mutation and non PAM-derived melanoma. This difference needs to be clarified by testing larger cohorts. The presence of a *TERT* promoter mutation in the tumor could have important clinical consequences, including the correlation of mutation status of this gene and follow-up. We found a correlation between the presence of a *TERT* promoter mutation and MFS, with a lower MFS in patients with a CM with a *TERT* promoter mutation, congruent with the findings in our previous study [13]. *TERT* promoter mutations have also been described as an independent prognostic factor in cutaneous melanoma. From this perspective, it is important to mention that most lesions in our cohort concerned relatively large tumors located at prognostic adverse locations (palpebra, fornix, or caruncle) [6], suggesting a bias. Patients with a *TERT*-promoter-mutated CM might benefit from an intensified follow-up program. 

In addition to *TERT* promoter mutations, CM frequently harbors *BRAF* mutations, which are known to activate the downstream kinases MEK1/2 and ERK1/2, resulting in tumor proliferation [1,6]. In this study, we identified *BRAF* mutations in almost half the cases, almost all resulting in V600E mutations. This is in line with the literature in which 30–40% of all CM harbor mutations in *BRAF*, almost all being V600E mutations [3,6,13,27,28]. These mutations, and specifically the V600E mutation, are also present in about half of all patients with cutaneous melanoma [29], whereas this mutation is not frequently involved in other mucosal melanoma or UM [6]. 

In cutaneous melanoma, the presence of a *TERT* promoter mutation in addition to a *BRAF* mutation is associated with unfavorable clinicopathological characteristics, such as large tumor thickness and a high mitotic rate [26]. Unfortunately, the number of cases in the current cohort was too small to render any conclusions concerning these correlations in CM. 

Determining the mutation status of the tumor could be useful with regards to therapeutic consequences, since several studies have shown an improved progression-free survival and overall survival, in patients with metastasized cutaneous melanoma harboring a *BRAF* mutation, using BRAF inhibitors [30]. *BRAF* mutations are also attractive as a target for adjuvant therapy in CM [6,31,32,33].

*NRAS* mutations are described in 27% of cutaneous melanoma, with a Q61K mutation as the most common mutation followed by Q61R [34]. *NRAS*-mutated cutaneous melanoma have an unfavorable prognosis as compared to *BRAF* mutated or wild-type melanoma [34]. We identified *NRAS* mutations in 21% of all CM in our cohort, which is in line with the 17% previously reported [15] and is somewhat lower compared to other literature [6]. Due to the small numbers of *NRAS*-mutated cases in our cohort, no correlations to prognosis could be determined. *NRAS* mutations are mutually exclusive with *BRAF* mutations [6]. *NRAS* mutations are amenable to *MEK* inhibitor therapy, as has been shown for cutaneous melanoma [35]. *MEK* inhibitors reduce the growth of *NRAS* mutant CM cell lines [1]. As yet, no cases of *NRAS*-mutated metastatic melanoma treated with *MEK* inhibitors have been published.

Interestingly, we detected an *SF3B1* mutation at the hotspot R625, which is well-known in UM [3,28], and was reported in one CM case. The presence of a *SF3B1* mutation was reported previously in CM, however, this concerned a p.C1123Y mutation and not a hotspot mutation [36], and another study reported a missense mutation [15]. Although R625 *SF3B1* mutations are very rare in most melanoma, they have been identified in UM, including iris melanoma [19], and are less frequent in cutaneous melanoma as well as in vulvovaginal mucosal melanoma [36,37,38,39]. The occurrence of *SF3B1* mutations in mucosal melanoma other than CM is higher, with a prevalence of 42% and hotspot mutations in 30–37% [39,40]. The clinical significance of this mutation in CM is unknown, whereas in UM, *SF3B1* mutation is correlated to late metastatic disease [41]. The CM with this mutation was treated with excision. This case also included PAM and showed local recurrence, three and eight years after primary treatment. No metastasis developed in the follow-up period of 6.8 years. However, metastasis in *SF3B1*-mutated UM was described even after 10 years [41].

The CM cases in our cohort also harbored mutations in c-*KIT*, *PTEN*, and *BAP1*. These findings of mutations in c-*KIT*, *NRAS*, and *PTEN* are congruent with other literature [1,6], with c-*KIT* mutations reported in 39% of mucosal melanoma and being feasible for targeted therapy [42]. Of interest is the finding of mutations in *BAP1*, which is a common hemizygous mutation in UM [12,43]. *BAP1* is a tumor suppressor gene and individuals with cutaneous melanocytic neoplasm with a germline *BAP1* mutation, often have *BRAF* mutations, with these lesions reported to have a benign clinical course [43]. However, UM with somatic *BAP1* mutations are correlated to loss of chromosome 3 and early metastatic disease. CM has also been described in a patient with the *BAP1* tumor predisposition syndrome [44]. We identified heterozygous *BAP1* mutations that can be explained as passenger mutations without consequences, due to expression of the remaining non-affected allele. 

The genetic profile of CM differs from UM, another subtype of ocular melanoma, in which mutations in *GNAQ*/*GNA11* are frequently described [45]. In this study, none of the CM harbored an activating hotspot mutation in *GNAQ* or *GNA11.* These findings are congruent with other studies analyzing mutations in CM [15,46]. *BRAF* and *NRAS* mutations are extremely rare in UM [37]. Therefore, these mutations can be useful in distinguishing CM from UM. This may be of interest in the identification of the primary tumor site in the case of metastatic melanoma with unknown primary. It also warrants the need for exploration of the genetic background of metastatic melanocytic lesions. However, such molecular results need to be interpreted with care, since we describe *BAP1* and *SF3B1* mutations in CM in the current cohort.

We did not find a correlation concerning the presence of any of the mutations and the development of recurrent disease. Cases with recurrent disease harbored the most frequently found mutations only in a (very) low number of cases. This may imply that recurrence and metastasis relate to different molecular or physical processes.

In conclusion, based on our molecular findings, CM comprises a separate entity within the ocular melanoma group, although there certainly are overlapping molecular features with UM, such as the presence of *BAP1* and *SF3B1* mutations. This warrants careful interpretation of molecular data in the light of clinical findings. About three-quarter of CM contain drug-targetable mutations in *BRAF*, *NRAS*, or *c*-*KIT*, supporting the relevance of molecular genetic testing in CM for therapeutic reasons. Within this study, we confirmed that *TERT* promoter mutations are frequently found in CM and are correlated to metastatic disease, supporting the relevance of molecular genetic testing for prognostic reasons.

## 4. Materials and Methods

### 4.1. Material Selection

We collected twenty-eight CM, diagnosed between 1987 and 2016 at the Erasmus MC—University Medical Center (Rotterdam, The Netherlands) and The Rotterdam Eye Hospital (Rotterdam, The Netherlands). Ethics Committee approval was obtained by the Medical Ethics Committee, Erasmus MC-University Medical Center, Rotterdam, The Netherlands (4 October 2018) and was registered with reference 67865. The study was performed according to the tenets of the Declaration of Helsinki. Samples were included when sufficient FFPE material was available for testing. Data regarding gender, age at the time of diagnosis, location, tumor thickness, the origin of the lesion, and information of development of recurrences and metastasis were collected from the patient records and information was obtained from the pathology reports and the nationwide-pathology network and registry system (*Pathologisch-Anatomisch Landelijk Geautomatiseerd Archief*). Recurrence was defined as histopathological proven CM at the same location, either after complete excision of the primary lesion or a tumor-free mapping biopsy, after a first incomplete excision of the primary tumor. Recurrence-free survival was defined as the time from the primary treatment to the date of recurrence or last date of follow-up. Metastasis-free survival was defined as time from the primary treatment to the date of metastatic disease or last date of follow-up. 

### 4.2. DNA Isolation

DNA from FFPE tissue was isolated using lysis buffer (Promega, Madison, WI, USA) and 5% Chelex (Bio-Rad, Hercules, CA, USA), as described previously [27] and stored at −20 °C. DNA concentrations were measured with the Quant-iT™ PicoGreen™ ds DNA Assay Kit (Thermo Fisher Scientific, Inc., Waltham, MA, USA). 

### 4.3. Targeted Next-Generation Sequencing

The Ion Personal Genome Machine and Torrent Server (Thermo Fisher Scientific, Waltham, MA, USA) was used for targeted next-generation sequencing (NGS), according to the manufacturer’s protocol. An input of DNA was used depending on the available amount of DNA. An extended gene panel covering *GNAQ*, *GNA11*, *EIF1AX*, *SF3B1*, *BAP1*, *BRAF*, *NRAS*, *c-KIT*, *PTEN*, and *TERT* was used, as described previously [27]. 

### 4.4. Mutation Analysis

Mutation analysis was performed independently by an ophthalmology resident (NvP) and a fellow in ophthalmic pathology (JvI), trained in the evaluation of NGS data. All data were analyzed manually using Integrative Genomics Viewer (IGV) Version 2.3.68 (97) (Broad Institute, Cambridge, MA). Furthermore, an automatic filtering of the variant calling files (vcf) was done according to the following criteria—inclusion of the hotspots at *GNAQ*/*GNA11* (R183 and Q209) and *SF3B1* (R625), and other variants meeting the following criteria—coverage of at least 50 reads and an allele frequency of at least 10%. Single nucleotide pleomorphisms (SNP’s), synonymous, intergenic, and intronic variants were excluded, but intronic variants with possible splice effects were scored. Subsequently, the filtered mutations were verified using IGV (Broad Institute, Cambridge, MA, USA), and compared to the mutations that were detected manually. 

### 4.5. Immunohistochemistry

The presence of a mutation in the *BAP1* gene was also evaluated using *BAP1* immunohistochemistry, clone sc-28383, 1:50 dilution (Santa Cruz Biotechnology, Dallas, TX, USA). The samples were scored through masked screening, by an experienced ophthalmic pathologist (RVE).

### 4.6. Survival Analysis

All statistical analysis was performed using IBM SPSS Statistics Version 25 (IBM, Armonk, NY, USA). Kaplan Meier estimates were used to compare survival between groups. Log-rank test was used to test the null hypothesis that there was no difference in survival. A *p*-value < 0.05 was considered to be statistically significant. For the purpose of analyzing age related to the mutation, age was categorized into three groups: <50 years, 50–65 years, >65 years, analogous to other literature [28]. Fisher’s exact test was used to analyze whether a specific mutation was correlated with a specific clinical or histopathological parameter.

## Figures and Tables

**Table 1:** Clinical and histopathological characteristics of the included conjunctival melanoma (CM).

Clinical Characteristics	
**Median age at diagnosis (years)**	63 (16–89)
Gender	
Male	14 (50%)
Female	14 (50%)
Location	
Bulbar	16 (57%)
Palpebral	10 (36%)
Fornix	5 (18%)
Caruncle	1 (4%)
Metastasis	
No	19 (68%)
Yes	9 (32%)
Local recurrence	
No	20 (71%)
Yes	8 (29%)
**Histopathological Characteristics**	

Median diameter (cm)	0.7 (0.05–1.8)
Median tumor thickness (mm)	3.0 (0.18–7.70)
pT status	
pT1a	6 (21%)
pT1b	5 (18%)
pT2a	1 (4%)
pT2b	11 (39%)
pTx	5 (18%)
Origin	
PAM	18 (64%)
Nevus	4 (14%)
De novo	3 (11%)
Unknown	3 (11%)

Clinical and histopathological characteristics of the included conjunctival melanoma (CM) cases. PAM = primary acquired melanosis with atypia. pT status = pathological tumor status.

**Table 2:** Presence of a mutation versus metastasis-free survival (MFS) and recurrence-free survival (RFS).

Gene	Presence of a Mutation	n (%)	Metastasis n (%)	MFS *p*-Value	Recurrences n (%)	RFS *p*-Value
*SF3B1*				0.45		0.45
	Yes	1 (4)	0 (0)		0 (0)	
	No	27 (96)	9 (33)		8 (30)	
*BAP1*				0.46		0.69
	Yes	5 (18)	1 (20)		2 (40)	
	No	23 (82)	8 (35)		6 (26)	
*TERT*				**0.008**		0.20
	Yes	15 (54)	7 (47)		2 (13)	
	No	13 (46)	2 (15)		6 (46)	
*NRAS*				0.17		0.82
	Yes	6 (21)	4 (67)		2 (33)	
	No	22 (79)	5 (23)		6 (27)	
*KIT*				0.26		0.88
	Yes	2 (7)	0 (0)		1 (50)	
	No	26 (93)	9 (35)		7 (28)	
*PTEN*				0.53		0.25
	Yes	4 (14)	1 (25)		2 (50)	
	No	24 (86)	8 (33)		6 (25)	
*BRAF*				0.052		0.76
	Yes	13 (46)	5 (38)		2 (15)	
	No	15 (54)	4 (27)		6 (40)	

The total number of included conjunctival melanoma cases was twenty-eight. This table depicts the percentages of the specific mutations in the cohort, as well as the development of metastatic disease and recurrent disease within the group of a specific mutation. The statistically significant *p*-value is depicted in bold. MFS = metastasis-free survival. RFS = recurrence-free survival.

**Table 3:** Mutations versus clinical and histopathological parameters.

		*TERT* n = 15 (%)	*P*	*BRAF* n = 13 (%)	P	*BAP1* n = 5 (%)	P	*NRAS* n = 6 (%)	*p*	*PTEN* n = 4 (%)	P	*c-KIT* n= 2 (%)	*P*	*SF3B1* n = 1 (%)	P
Gender			0.26		0.71		1.00		1.00		1.00		1.00		1.00
	Male	6 (40)		6 (46)		3 (60)		3 (50)		3 (75)		1 (50)		1 (100)	
	Female	9 (60)		7 (54)		2 (40)		3 (50)		1 (25)		1 (50)		0 (0)	
Age			0.91		0.91		0.52		0.32		0.92		0.24		0.50
	<50y	2 (13)		2 (15)		0 (0)		2 (33)		0 (0)		0 (0)		0 (0)	
	50–65y	7 (47)		6 (46)		3 (60)		2 (33)		2 (50)		2 (100)		1 (100)	
	>65y	6 (40)		5 (38)		2 (40)		2 (33)		2 (50)		0 (0)		0 (0)	
Location			0.16		0.85		1.00		1.00		0.59		1.00		0.48
	Bulbar	8 (53)		6 (46)		2 (40)		2 (33)		1 (25)		1 (50)		1 (100)	
	Forniceal/ palpebral/ caruncular involvement	5 (33)		6 (46)		2 (40)		2 (33)		2 (50)		1 (50)		0 (0)	
Tumor thick-ness			0.67		0.68		1.00		0.63		0.56		0.53		0.31
	Tumor thickness ≤2 mm	5 (33)		3 (23)		1 (20)		2 (33)		2 (50)		1 (50)		1 (100)	
	Tumor thickness >2mm	8 (53)		9 (69)		4 (80)		3 (50)		2 (50)		1 (50)		0 (0)	
pT status			0.16		0.85		1.00		1.00		0.59		1.00		0.48
	pT1	8 (53)		6 (46)		2 (40)		2 (33)		1 (25)		1 (50)		1 (100)	
	pT2	5 (33)		6 (46)		2 (40)		2 (33)		2 (50)		1 (50)		0 (0)	
Origin			**0.01**		1.00		1.00		0.30		1.00		1.00		1.00
	PAM	6 (40)		7 (54)		3 (60)		3 (50)		3 (80)		2 (100)		1 (100)	
	Non PAM (nevus/de novo)	7 (47)		3 (23)		1 (20)		3 (50)		1 (25)		0 (0)		0 (0)	

P = *p*-value calculated with either the Pearson’s χ^2^ test or Fisher’s exact test. In bold, the association between the presence of a *TERT* promoter mutation and origin of the lesion (*p*-value = 0.01), with most cases (54%) developing either de novo or from a melanocytic nevus. None of the cases showed *GNAQ*, *GNA11*, or *EIF1AX* mutations; therefore, these mutations are not included in the table. pT status = pathological tumor status.

## Data Availability

Data can be provided upon request.

## Supplementary Materials

The following are available online at https://www.mdpi.com/article/10.3390/ijms22115784/s1. Supplementary Table S1: Overview of mutations detected in conjunctival melanoma.
