# Supplementary material for: Molecular Genetics of Conjunctival Melanoma and Prognostic Value of TERT Promoter Mutation Analysis
Source: Int J Mol Sci. 2021 May 28;22(11):5784. doi: 10.3390/ijms22115784 (PMC8198138; doi:10.3390/ijms22115784)
Supplement: Supplementary file 1 [file ijms-22-05784-s001.zip › ijms-1219237-supplementary.pdf]

	SF3B1	*BAP1*	*TERT*	*NRAS*	*KIT*	*PTEN*	*BRAF*
1	wt	wt	wt	wt	wt	c.675T>G: p.(Tyr225*)[#]	c.1799T>A: p.(Val600Glu)[*]
2	wt	wt	c.-146C>T[*]	wt	wt	wt	c.1799T>A: p.(Val600Glu)[*]
3	wt	wt	wt	wt	wt	wt	wt
4	wt	wt	wt	wt	wt	wt	wt
5	wt	wt	c.-146C>T[*]	wt	wt	wt	c.1799T>A: p.(Val600Glu)[*]
6	wt	wt	c.-146C>T[*]	wt	wt	wt	c.1799T>A: p.(Val600Glu)[*]
7	wt	wt	wt	wt	wt	wt	wt
8	wt	wt	c.-146C>T[*]	wt	wt	wt	c.1799T>A: p.(Val600Glu)[*]
9	wt	wt	wt	c.181C>A: p.(Gln61Lys)[*]	wt	wt	wt
10	wt	wt	c.-124C>T[*]	wt	wt	c.47dup: p.(Tyr16*)[#]	c.1799T>A: p.(Val600Glu)[*]
11	wt	wt	wt	wt	wt	wt	c.1799T>A: p.(Val600Glu)[*]
12	wt	wt	c.-146C>T[*]	c.181C>A: p.(Gln61Lys)[*]	wt	wt	wt
13	wt	c.1202_1203del: p.(Tyr401*)[$]	c.-146C>T[*]	c.182A>G: p.(Gln61Arg)[*]	wt	wt	wt
14	wt	wt	c.-138C>T[*]<br>c.-139C>T[*]	c.182A>G: p.(Gln61Arg)[*]	wt	wt	wt
15	wt	wt	c.-146C>T[*]	wt	wt	wt	c.1799T>A: p.(Val600Glu)[*]
16	wt	wt	wt	wt	wt	wt	c.1798G>A + c.1799T>A :p.(Val600Lys)[*]
17	wt	c.1144G>A: p.(Gly382Ser)[$]	wt	wt	c.1144G>A: p.(Gly382Ser)[*]<br>c.2435G>T: p.(Gly812Val)[*]	c.133G>A: p.(Val45Ile)[#]<br>c.701G>A: p.(Arg234Gln)[#]	wt
18	wt	wt	wt	wt	wt	wt	wt
19	wt	wt	c.-146C>T[*]	wt	wt	wt	c.1799t>a: p.(Val600Glu)[*]
20	wt	wt	c.-113C>T[*]<br>c.-124C>T[*]	wt	wt	wt	wt
21	wt	wt	wt	wt	wt	wt	wt
22	c.1874G>A:p.(Arg625His)[&]	wt	wt	wt	c.1697_1720del:p.(Asn566_Pro573del)[*]	wt	wt
23	wt	wt	c.-146C>T[*]	wt	wt	wt	c.1799T>A: p.(Val600Glu)[*]
24	wt	wt	c.-146C>T[*]	wt	wt	wt	c.1799T>A: p.(Val600Glu)[*]<br>c.1362T>A:p.(Asp454Glu)[*]
25	wt	wt	c.-146C>T[*]	c.183A>T:p.(Gln61His)[*]	wt	wt	wt
26	wt	c.1729+2T>A[$]	wt	c.34G>C:p.(Gly12Arg)[*]	wt	wt	wt
27	wt	c.1208A>G:p.(Asp403Gly)[$]	c.-146C>T[*]	wt	wt	wt	c.1796_1797insCAT:p.(Thr599_Val600insIle)[*]
28	wt	c.935G>A:p.(Gly312Asp)[$]	wt	wt	wt	c.634+1G>A[#]	wt

wt=wildtype, *gain of function, # loss of function, &alternative splicing, $ passenger mutation
